# Suitability of Artificial Diets Containing Various Types of Pollen Grains to *Helicoverpa armigera* (Hübner, 1808): Nutritional Performance and Digestive Enzyme Response

**DOI:** 10.3390/insects16040429

**Published:** 2025-04-19

**Authors:** Fatemeh Kefayat, Seyed Ali Hemmati, Arash Rasekh, Fatemeh Nasernakhaei, Lukasz L. Stelinski

**Affiliations:** 1 Department of Plant Protection, Faculty of Agriculture, Shahid Chamran University of Ahvaz, Ahvaz 61357-43311, Iran; fateme.kefayat@gmail.com (F.K.); a.rasekh@scu.ac.ir (A.R.); 2 Department of Genetic and Plant Production, Faculty of Agriculture, Shahid Chamran University of Ahvaz, Ahvaz 61357-43311, Iran; f.nasernakhaei@scu.ac.ir; 3 Department of Entomology and Nematology, Citrus Research and Education Center, University of Florida, Lake Alfred, FL 33850, USA; stelinski@ufl.edu

**Keywords:** cotton bollworm, feeding efficiency, digestive enzymes, mass rearing, nutritional value

## Abstract

The development of an effective artificial diet is essential for the mass rearing of insects used in pest management programs, including augmentative biological control, insecticide resistance monitoring, and sterile insect release. This study evaluated the consumption, utilization, and enzymatic responses of the polyphagous pest *Helicoverpa armigera* (Hübner, 1808) (Lepidoptera: Noctuidae) when reared on meridic diets supplemented with different pollen grains. The control diet followed a well-established meridic formulation, while the eight experimental diets incorporated pollen from the honey bee, rapeseed, maize, sunflower, hollyhock, glossy shower, saffron, and date palm. The findings indicate that pollen supplementation enhances the quality of artificial diets for *H. armigera*. Larvae fed on the date palm pollen-supplemented diet exhibited significantly higher weight gain, efficiency of conversion of ingested food (ECI), efficiency of conversion of digested food (ECD), and relative growth rate (RGR) compared to those fed on the control diet. The highest relative consumption rate (RCR) was observed in larvae fed on the sunflower pollen-supplemented diet. Additionally, pollen-supplemented diets influenced the amylolytic and proteolytic enzyme activities of *H. armigera* larvae in a diet-dependent manner. Nutritional analysis of the pollen types revealed significant variations in the sugar, lipid, and protein contents. Cluster analysis further identified the date palm pollen-supplemented diet as the most nutritionally beneficial, suggesting its potential application in the large-scale production of *H. armigera*.

## Simple Summary

*Helicoverpa armigera* (Hübner, 1808) (Lepidoptera: Noctuidae) is a polyphagous pest of significant agricultural importance. This study investigated the variation in food consumption, nutrient utilization, and digestive enzyme activity in *H. armigera* larvae when fed diets supplemented with various pollen types. In addition, the nutritional composition—specifically sugar, lipid, protein, and total phenolic contents—of the pollen grains was analyzed to explore potential correlations between the insect’s feeding performance and the biochemical characteristics of the pollen. Experimental diets were prepared by supplementing a standard meridic diet (used as the control) with pollen grains from honey bees (*Apis mellifera*), rapeseed (*Brassica napus*), maize (*Zea mays*), sunflower (*Helianthus annuus*), hollyhock (*Alcea* spp.), glossy shower (*Cassia glauca*), saffron (*Crocus sativus*), and date palm (*Phoenix dactylifera*). The biochemical properties of the pollen significantly influenced larval growth, feeding efficiency, and the activity of digestive enzymes in *H. armigera*. Among the tested treatments, the diet containing date palm pollen demonstrated the most promising results, suggesting its potential utility in enhancing mass-rearing protocols for integrated pest management strategies.

## 1. Introduction

In augmentative biological control, natural enemies are released in large numbers to suppress target insect populations [1]. However, sustaining the mass production of these beneficial organisms requires the large-scale rearing of both the natural enemy and its host or prey [1,2]. Over the past century, the mass production of natural enemies has been facilitated by the development of artificial diets for insect pests, particularly lepidopterans [3]. Several economically significant species of Lepidoptera serve as primary or secondary (intermediate) hosts for the mass production of natural enemies [4,5,6], including *Helicoverpa armigera* (Hübner, 1808) (Lepidoptera: Noctuidae), commonly known as the cotton bollworm. This polyphagous agricultural pest feeds on approximately 170 plant species worldwide [7], causing significant yield losses by consuming reproductive structures such as flowers and fruits [7]. Despite its status as a pest, *H. armigera* is extensively reared in laboratory settings for research purposes, including studies on feeding behavior, life table parameters, physiological responses, and toxicology [8,9,10]. Furthermore, mass-reared individuals of *H. armigera*, in the form of eggs and larvae, are used in insect breeding facilities to support the large-scale production of natural enemies such as *Trichogramma* and *Bracon* wasps [11,12].

The development of more efficient and cost-effective methods is crucial for the large-scale production of insects [13]. One of the most promising approaches for rearing phytophagous insects, particularly *H. armigera*, is the use of artificial diets [1]. Compared to rearing on natural host plants, artificial diets offer several advantages, including improved growth rates, enhanced population uniformity, year-round rearing capability, and reduced labor costs [13,14]. Given the broad host range of *H. armigera*, numerous artificial diets have been formulated to support its continuous rearing [9,10,15,16]. The formulation of an optimal artificial diet requires an understanding of both the insect’s nutritional requirements and the biochemical composition of its natural food sources. Insects have specific dietary needs essential for growth, development, and reproduction, particularly a high demand for protein, which they obtain from protein-rich food sources [10,17]. In addition to proteins, other essential nutrients, including carbohydrates, lipids, vitamins, and minerals, play vital roles in insect development, energy metabolism, and physiological functions and must be carefully incorporated into artificial diets [16]. Several plant-based protein sources, such as broad beans, kidney beans, cowpeas, common beans, and corn, have been used in artificial diets formulated for noctuid species [8,16,18]. Additionally, efforts have been made to enrich insect diets with alternative natural protein sources, including bee pollen and other pollen types, to enhance diet quality and nutritional balance [2,19,20,21].

Pollen, produced by both monocotyledonous and dicotyledonous plants, consists of male gametes that fertilize female ovules, leading to seed formation [22]. The pollen grain is enclosed by a multilayered wall, with the outer exine composed primarily of phenolic compounds and fatty acids, while the inner intine is associated with polysaccharide metabolism [23]. The cytoplasm of pollen grains is rich in nutrients, with protein being the predominant component. The protein content varies among plant families, ranging from 15–25% in *Onagraceae* and *Cactaceae* to over 50% in *Solanaceae*, *Melastomataceae*, and *Cochlospermataceae* [24,25]. In addition to protein, pollen contains substantial amounts of carbohydrates (primarily starch), lipids (mainly sterols), and essential minerals, including carbon, oxygen, magnesium, phosphorus, potassium, calcium, molybdenum, and aluminum [26,27]. Bee pollen, which results from the agglutination of pollen grains from various plant species with honey bee gland secretions and nectar, serves as a highly nutritious food source, being particularly rich in protein for insects [28]. Riahi et al. (2016) analyzed the nutritional composition of pollen from several plant species, including almond, bitter orange, sunflower, date palm, maize, bee pollen, and castor bean, and reported significant variation in the protein, sugar, and lipid contents among species [29]. Due to its rich nutritional profile, pollen consumption can enhance the survival of immature insect stages and increase progeny production in adults [30]. Pollen has also been explored as an alternative or supplementary food source for predatory insects, supporting their establishment in the absence or scarcity of prey. For instance, date palm pollen [31] and cattail pollen [32] have been shown to promote the feeding and growth of phytoseiid mite individuals. Recent studies have also investigated the incorporation of pollen into artificial diets for insect rearing, demonstrating that pollen supplementation enhances the diet’s capacity to support consistent mass production across multiple generations [2,33]. For example, Maruccia et al. (2019) reared *Doru luteipes* (Scudder, 1876) (Dermaptera: Forficulidae) on an artificial diet supplemented with maize pollen and observed a significant improvement in insect performance [30].

To the best of our knowledge, pollen has primarily been utilized in the rearing of biological control agents, and its application in the rearing of insect pests such as *H. armigera* remains limited [29,30,31]. Given that pollen alone does not provide sufficient protein to support optimal growth in *H. armigera* and considering the variability in its nutritional composition across plant species, this study investigated the effects of incorporating a diverse range of pollen types into a basal artificial diet. The formulated diet included cowpea (*Vigna sinensis* L.) seed powder as the principal protein source and was supplemented with bee pollen and pollen from various plant species to evaluate their influence on diet consumption and nutrient utilization by *H. armigera* larvae. In addition, the physiological responses to the pollen-supplemented diets were assessed by measuring the activities of key digestive enzymes, specifically amylases and proteases. The biochemical composition of each pollen type—including protein, sugar, lipid, and total phenolic contents—was also analyzed to determine its nutritional potential in artificial diets. The results of this study contribute valuable insights toward the optimization of artificial diets for the efficient mass rearing of *H. armigera* larvae, with implications for both research and pest management applications.

## 2. Materials and Methods

### 2.1. Pollen Collection

Pollen grains from seven plant species—rapeseed (*Brassica napus* L.), maize (*Zea mays* L.), sunflower (*Helianthus annuus* L.), hollyhock (*Alcea ficifolia* L.), glossy shower (*Senna surattensis* (Burm. f.) H. S. Irwin & Barneby), saffron (*Crocus sativus* L.), and date palm (*Phoenix dactylifera* L.)—along with honey bee (*Apis mellifera* L.)-harvested pollen, were used in this study. These pollen grains were chosen because their associated plant species are found extensively in Khuzestan Province, Iran. The only exception was saffron pollen grain, which was previously shown to have high nutritional content [34]. The pollen grains were collected from pesticide-free plants in Khuzestan Province, except for saffron pollen, which was sourced from South Khorasan Province, Iran. Date palm pollen was obtained from the Date Palm and Tropical Fruits Research Institute in Ahvaz, Iran, while honey bee pollen, which was a mixture of pollen grains from different plants, was supplied by local beekeepers in Ahvaz, Khuzestan Province, Iran. The remaining pollen types were directly collected from the reproductive organs of the respective plants by shaking or brushing them into paper bags. After collection, the pollen grains were transported to the laboratory, dried at room temperature, and sieved to remove impurities. The processed pollen grains were stored at 4 °C for short-term use or at −20 °C for long-term storage (less than 3 months) before incorporation into artificial diets.

### 2.2. Insect Source and Rearing

The *H. armigera* population used in this study was initially collected as larvae from unsprayed bean fields in Khuzestan Province, Iran. Young larvae (first to third instars) were reared in groups, while later instars were housed individually in Petri dishes (9 cm in diameter) to prevent cannibalism, with net-covered openings in the lids of all dishes to ensure air ventilation. The larvae were fed an artificial diet [35], which was replaced as needed. Once the larvae ceased feeding, they were collected and individually transferred to pupation containers (3 × 7 cm), providing sufficient space for full wing expansion after adult emergence. Upon eclosion, ten pairs of adults were placed in oviposition containers (15 × 25 cm) lined with paper sheets as egg-laying substrates. The containers were covered with a mesh net to allow for proper aeration. Adults were provided with a 10% honey solution as a food source, which was replenished daily. The *H. armigera* individuals were maintained in an environmentally controlled growth chamber at 25 ± 1 °C, 65 ± 5% relative humidity, and a 16L:8D photoperiod. Experimental procedures were conducted using the F2 generation.

### 2.3. Preparation of Artificial Diets with Different Pollen Grains

Nine different artificial diets were prepared and evaluated. The control diet was a well-established meridic formulation based on the formulation developed by Shorey and Hale (1965) [35], while the other eight diets were modified versions incorporating different pollen grains, specifically from the honey bee, rapeseed, maize, sunflower, hollyhock, glossy shower, saffron, and date palm. Each modified diet was named after the pollen it contained. The modification involved reducing the amount of cowpea (*Vigna unguiculata* L. Walp.) seed powder from 20.5 g to 19.5 g and substituting 1 g (chosen based on our preliminary optimization) of the respective pollen grain while keeping all other ingredients unchanged. The compositions and quantities of all diet components are provided in Table 1.

For diet preparation, large pollen grains, such as those from rapeseed, glossy shower, and honey bee pollen pellets, were first ground before incorporation, whereas smaller pollen grains were used directly. All ingredients, including the pollen grains, were weighed and thoroughly mixed before being combined with distilled water containing pre-weighed agar maintained at 40–50 °C. The mixture was homogenized using a handheld glass homogenizer for 5 to 10 min to achieve a semi-solid consistency and then cooled to room temperature before adding antimicrobial compounds (methyl *p*-hydroxybenzoate and formaldehyde). Fresh diets were prepared weekly and stored under refrigeration until use. Before feeding, refrigerated diets were conditioned to room temperature for 2 to 3 h.

### 2.4. Diet Consumption and Utilization

To assess the effects of pollen-containing diets on the nutritional performance of *H. armigera*, third-instar larvae (n = 25) from synchronized egg batches were used for each dietary treatment. The larvae were individually placed in 9 cm diameter Petri dishes with net-covered openings in the lids for aeration and provided with appropriate amounts of the respective pollen-containing diets. The diets were replaced daily with fresh portions (1 g), and uneaten food was removed. Larval weights were recorded before and after feeding using an analytical balance (Sartorius AG Germany GCA803S, Göttingen, Germany, d = 0.001 ct) until the pre-pupal stage. Additionally, the weight of the diet provided, diet remaining, and frass produced were measured daily. Pre-pupal and pupal weights were also recorded for larvae reared on each pollen-containing diet. Since nutritional indices are calculated based on dry weight, samples of *H. armigera* larvae, frass, and offered food (n = 20 per diet) were weighed, oven-dried at 60 °C for 48 h, and reweighed to determine their dry weight percentages. The diet consumption and utilization efficiency of *H. armigera* larvae were calculated using the following formulae [36]:

(1) $$\mathrm{Consumption}\;\mathrm{index}\;\left(\mathrm{CI}\right)=\frac{E}{A}$$

(2) $$\mathrm{Approximate}\;\mathrm{digestibility}\;\left(\mathrm{AD}\right)=\frac{E-F}{E}$$

(3) $$\mathrm{Efficiency}\;\mathrm{of}\;\mathrm{conversion}\;\mathrm{of}\;\mathrm{ingested}\;\mathrm{food}\;\left(\mathrm{ECI}\right)=\frac{P}{E}\times 100$$

(4) $$\mathrm{Efficiency}\;\mathrm{of}\;\mathrm{conversion}\;\mathrm{of}\;\mathrm{digested}\;\mathrm{food}\;\left(\mathrm{ECD}\right)=\frac{P}{E-F}\times 100$$

(5) $$\mathrm{Relative}\;\mathrm{consumption}\;\mathrm{rate}\;\left(\mathrm{RCR}\right)=\frac{E}{W_{0}\times T}$$

(6) $$\mathrm{Relative}\;\mathrm{growth}\;\mathrm{rate}\;\left(\mathrm{RGR}\right)=\frac{P}{W_{0}\times T}$$

where *E* is the dry weight of food consumed (mg), *A* is the average larval dry weight over time (mg), *F* is the dry weight of frass produced (mg), *p* is the dry weight gain of larvae (mg), *W*_0_ is the primary weight of larvae (mg), and *T* is the feeding duration (day). Moreover, weight gain was calculated as the difference between the final larval weight and the weight at the beginning of the third instar. The weight of food consumed was quantified as the difference between the weight of newly served diet and that remaining after 24 h.

### 2.5. Analysis of Midgut Enzymes

Midgut enzyme extraction was performed on late-instar *H. armigera* larvae (n = 20), which were randomly selected from the same pollen-containing artificial diet on which they had been previously reared. Each larva was dissected in ice-cold distilled water under a stereomicroscope (Stemi SV6, ZEISS, Oberkochen, Germany), and the midgut was carefully removed. The midguts were processed in distilled water using a handheld homogenizer and subsequently centrifuged at 16,000× *g* at 4 °C for 10 min. The resulting supernatants were pooled and stored in 1.5 mL screw-cap microtubes at −20 °C for further analysis [37,38].

Amylase activity in late-instar *H. armigera* larvae fed on the pollen-containing diets was measured following the protocol described by Bernfeld (1955) [39]. Enzyme assays were conducted by incubating *H. armigera* enzyme samples (10 μL) with a universal buffer system (50 μL, succinate–glycine–2-morpholinoethanesulfonic acid) and soluble starch (1%, 20 μL) as a substrate at 35 °C for 10 min. The reaction was terminated by adding 3,5-dinitrosalicylic acid (DNSA) reagent (100 mL), followed by heating in boiling water for 10 min. Absorbance was then measured spectrophotometrically at 540 nm (S2100SUV, UNICO, Tucson, AZ, USA). All assays were performed in triplicate with corresponding blanks. Amylase activity was expressed as the amount of enzyme required to produce 1 mg of maltose at 37 °C within 30 min.

General proteolytic activity in the midgut of late-instar *H. armigera* larvae fed on the tested diets was assessed using azocasein as a protein substrate [40]. Midgut extracts (50 μL) were incubated with an azocasein solution (1.5%, 80 μL) prepared in a universal buffer (50 mM) at 37 °C for 50 min. Proteolysis was terminated by adding 30% trichloroacetic acid (TCA) (100 μL) to the reaction mixture. The unhydrolyzed azocasein was removed by incubating the mixture at 4 °C for 30 min, followed by centrifugation at 16,000 g for 10 min. The resulting supernatant was mixed with an equal volume of NaOH (1 M), and absorbance was measured at 440 nm using a UV spectrophotometer (S2100SUV, UNICO, Tucson, AZ, USA). Blank assays were conducted under the same conditions, replacing the enzyme extract with distilled water. Each assay was performed in triplicate for each pollen-containing diet. One unit of protease activity was defined as the increase in optical density per milligram of protein in the enzyme sample per minute due to azocasein proteolysis.

### 2.6. Biochemical Analysis of Pollen Grains

The total crude protein content of various pollen grains was determined using the Kjeldahl method, with protein content estimated by multiplying the nitrogen content by 6.25 [41]. Sugar content was measured using the phenol–sulfuric acid assay, where the sample was mixed with sulfuric acid and a phenol solution. The resulting color complex was quantified via photometric measurement [42]. The total lipid content of the tested pollen grains was quantified using a Soxhlet extractor with n-hexane as the solvent, and the extract weight was determined gravimetrically as crude lipid [34]. Phenolic content in the pollen grains was determined using the Folin–Ciocalteu method, with gallic acid as the standard. In this procedure, the pollen extract (500 µL), Folin–Ciocalteu reagent (500 µL), and sodium carbonate (Na_2_CO_3_) solution (10%, 500 µL) were mixed and incubated at room temperature for 1 h. Absorbance was then measured at 700 nm (S2100SUV, UNICO, Tucson, AZ, USA). The phenolic content was expressed as milligrams of gallic acid equivalents per gram of pollen extract, based on a standard curve [43].

### 2.7. Data Analysis

Larval nutritional indices and enzymatic activities, along with the biochemical composition of pollen grains, were analyzed using one-way analysis of variance (ANOVA). Prior to analysis, data were assessed for normality using the Kolmogorov–Smirnov test. Tukey’s Honest Significant Difference (HSD) tests were employed to determine differences among the pollen-containing diets, with significance set at *p* < 0.01. Pearson’s correlation tests were used to explore the relationship between the nutritional and physiological characteristics of *H. armigera* and the biochemical traits of the various pollen grains. A dendrogram of the evaluated pollen-containing artificial diets, based on nutritional and growth indices as well as enzymatic activities of *H. armigera*, was constructed using Ward’s method. All analyses were performed using SPSS v. 22.0.

## 3. Results

### 3.1. Diet Consumption and Utilization

Significant differences were observed among the pollen-supplemented diets in terms of the nutritional parameters of *H. armigera* larvae. The consumption index (CI) was highest in larvae reared on the sunflower pollen-supplemented diet and lowest in those fed on the date palm pollen-supplemented diet (*F*_8,216_ = 11.853, *p* < 0.001; Table 2). The approximate digestibility (AD) was highest in larvae fed on the glossy shower pollen-supplemented diet, while the lowest values were recorded for those reared on honey bee and hollyhock pollen-supplemented diets (*F*_8,216_ = 13.265, *p* < 0.001; Table 2). The highest efficiency of conversion of ingested food (ECI) (*F*_8,216_ = 32.165, *p* < 0.001) and efficiency of conversion of digested food (ECD) (*F*_8,216_ = 31.700, *p* < 0.001) were recorded in larvae fed on the date palm pollen-supplemented diet, whereas the lowest values for both parameters were observed in those fed on the control diet (Table 2). The relative consumption rate (RCR) was highest in larvae reared on the sunflower pollen-supplemented diet and lowest in those fed on the control diet (*F*_8,216_ = 16.333, *p* < 0.001; Table 2). The relative growth rate (RGR) was highest in larvae reared on the date palm pollen-supplemented diet, whereas the lowest RGR values were observed in larvae fed on the glossy shower pollen-supplemented and control diets (*F*_8,216_ = 20.276, *p* < 0.001; Table 2).

**Figure 1:**
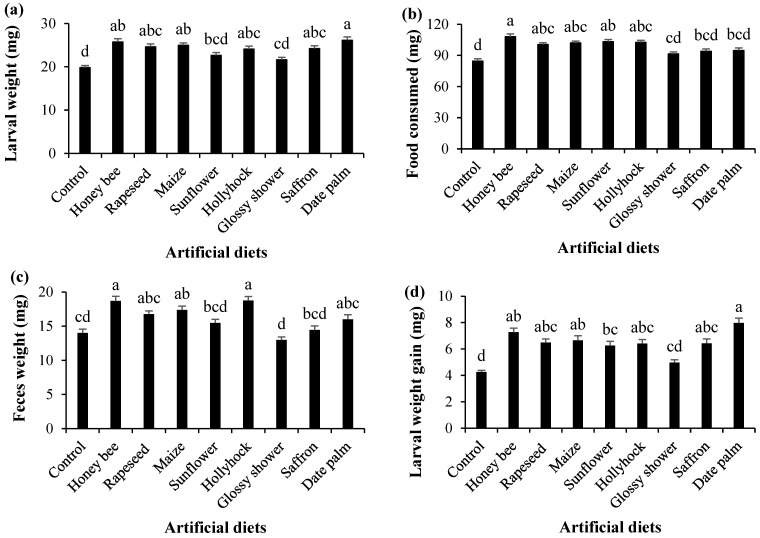
(**a**) Mean (±SE) larval weight, (**b**) food consumed, (**c**) frass weight, and (**d**) larval weight gain of *Helicoverpa armigera* reared on various pollen-containing artificial diets. Columns with different letters represent significant differences among treatments (Tukey’s HSD test, *p* < 0.01).

**Figure 2:**
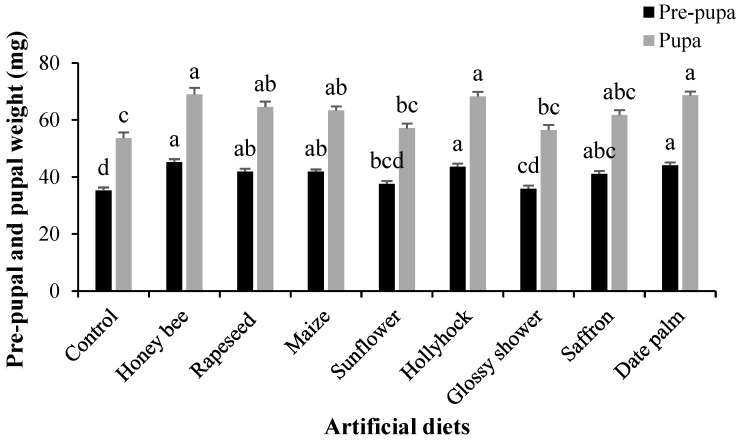
Mean (±SE) pre-pupal and pupal weight of *Helicoverpa armigera* reared on various pollen-containing artificial diets. Columns with different letters represent significant differences among treatments (Tukey’s HSD test, *p* < 0.01).

Significant differences were observed among the pollen-supplemented diets in terms of larval weight, food consumption, frass weight, larval weight gain, pre-pupal weight, and pupal weight (Figure 1 and Figure 2). *Helicoverpa armigera* larvae attained the highest weight when reared on the date palm pollen-supplemented diet, whereas the lowest larval weight was recorded on the control diet without pollen (*F*_8,216_ = 10.414, *p* < 0.001; Figure 1a). Food consumption was highest in larvae fed on the honey bee pollen-supplemented diet and lowest in those reared on the control diet (*F*_8,216_ = 10.991, *p* < 0.001; Figure 1b). Frass weight was highest in larvae fed on the honey bee and hollyhock pollen-supplemented diets, while the lowest frass weight was recorded on the glossy shower pollen-supplemented diet (*F*_8,216_ = 12.457, *p* < 0.001; Figure 1c).

The highest larval weight gain was observed in larvae reared on the date palm pollen-supplemented diet, whereas the lowest was recorded on the control diet (*F*_8,216_ = 13.771, *p* < 0.001; Figure 1d). Similarly, the pre-pupal weight (*F*_8,216_ = 11.621, *p* < 0.001) and pupal weight (*F*_8,216_ = 10.505, *p* < 0.001) were significantly higher in larvae fed on honey bee, hollyhock, and date palm pollen-supplemented diets, while the lowest values were recorded in those reared on the control diet (Figure 2).

### 3.2. Analysis of Midgut Enzymes

**Figure 3:**
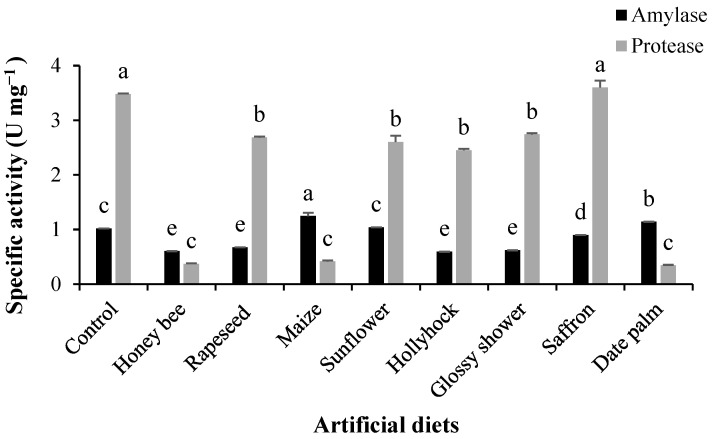
Mean (± SE) general amylolytic and proteolytic activities of midgut extracts from *Helicoverpa armigera* larvae reared on various pollen-containing artificial diets. Columns with different letters represent significant differences among treatments (Tukey’s HSD test, *p* < 0.01).

The activity of midgut enzymes in *H. armigera* larvae varied significantly among the pollen-supplemented diets (Figure 3). Amylolytic activity was highest in larvae reared on the maize pollen-supplemented diet, whereas the lowest activity was recorded in those fed on honey bee, rapeseed, hollyhock, and glossy shower pollen-supplemented diets (*F*_8,18_ = 24.146, *p* < 0.001; Figure 3). General proteolytic activity was highest in larvae reared on the control and saffron pollen-supplemented diets, while the lowest activity was observed in those fed on honey bee, maize, and date palm pollen-supplemented diets (*F*_8,18_ = 48.177, *p* < 0.001; Figure 3).

### 3.3. Pollen Grain Biochemical Analysis

The contents of protein, sugar, lipid, and phenol varied significantly among the studied pollen grains (Table 3). The protein content ranged from 9.520% in saffron pollen to 26.430% in date palm pollen (*F*_7,16_ = 7.170, *p* < 0.001; Table 3). The sugar content was highest in glossy shower pollen, while the lowest amount was found in rapeseed pollen (*F*_7,16_ = 5.956, *p* < 0.001; Table 3). The lipid content was highest in rapeseed pollen and lowest in honey bee pollen (*F*_7,16_ = 3.747, *p* < 0.001; Table 3). The total phenolic concentration ranged from 44.290 to 102.923 µg/g across the tested pollen grains, with sunflower pollen exhibiting the highest phenolic content and date palm pollen the lowest (*F*_7,16_ = 2.222, *p* < 0.001; Table 3).

### 3.4. Correlation Analysis

The correlation coefficients between feeding and physiological traits of *H. armigera* and the biochemical composition of different pollen grains are presented in Table 4. Larval and pupal weights exhibited significant negative correlations with sugar and total phenolic contents (*p* < 0.01). Significant positive correlations were found between the efficiency of conversion of ingested food (ECI), efficiency of conversion of digested food (ECD), and relative growth rate (RGR) with protein content across the various pollen grains (*p* < 0.01). Conversely, these indices showed significant negative correlations with sugar and phenolic contents (*p* < 0.01). Amylolytic activity was negatively correlated with lipid and total phenolic contents (*p* < 0.01). Proteolytic activity was negatively correlated with protein content (*p* < 0.01) while being positively correlated with lipid and total phenolic contents (*p* < 0.01). Neither food consumption nor the relative consumption rate (RCR) showed any significant correlation with the biochemical traits of the different pollen grains (*p* > 0.05).

### 3.5. Cluster Analysis

**Figure 4:**
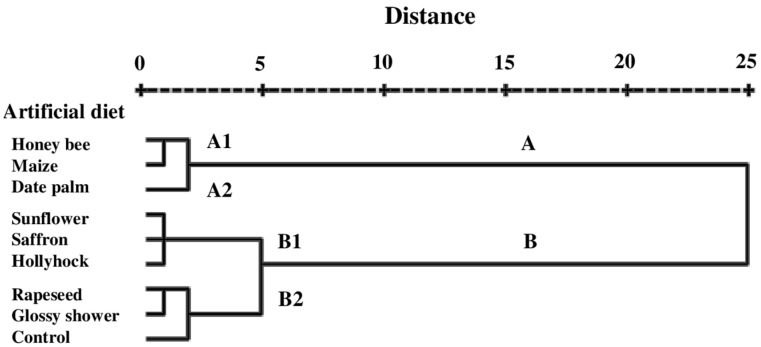
Dendrogram representing degree of relatedness of different pollen grains based on nutritional and growth indices and enzymatic activity of *Helicoverpa armigera* reared on various pollen–contained artificial diets (based on Ward’s method).

The dendrogram of feeding parameters and enzyme activity in *H. armigera* larvae reared on various pollen-supplemented diets revealed two main clusters, labeled A and B (Figure 4). Cluster A consisted of two sub-clusters: A1, which included the honey bee and maize pollen-supplemented diets, and A2, which included the date palm pollen-supplemented diet (Figure 4). Cluster B comprised sub-clusters B1 (sunflower, saffron, and hollyhock pollen-supplemented diets) and B2 (rapeseed and glossy shower pollen-supplemented diets and the control diet) (Figure 4).

## 4. Discussion

Our results demonstrated that all pollen grain types investigated were nutritious to *H. armigera* larvae, and their addition to the basic artificial diet formulation met the nutritional requirements of the larvae. Similar results were reported for other insect species using pollen from various plant species [29,31,33]. However, the key nutritional parameters and physiological responses of larvae were differentially influenced by the pollen-supplemented diets. A comparison of nutritional parameters revealed that larvae fed on the control diet consumed the least food and exhibited the lowest weight gain. Decreased food consumption is associated with an increased efficiency of conversion into body mass [44]. In contrast, the highest weight gain was observed in larvae reared on the date palm pollen-supplemented artificial diet. This could be attributed to the high protein content and low sugar, lipid, and total phenolic contents of the date palm pollen. While sugar and lipid contents are key indicators of the nutritional quality of pollen grains [45], it appears that *H. armigera* larvae preferentially consumed pollen grains with lower levels of sugar and lipids. The range of larval weight gain observed in this study was lower than those reported for *H. armigera* larvae fed on sunflower seed-based artificial diets [10] and legume seed-based artificial diets [16]. This discrepancy may be explained by genetic variation within the *H. armigera* population, differences between larval instars, and the type of seeds used in these studies compared to those examined in the present study.

Larvae fed on diets containing either honey bee pollen or hollyhock pollen exhibited the lowest assimilation efficiency (AD). Low AD values are often associated with sub-optimal nutritional content or high levels of crude fiber in proportion to nutrients in the consumed diet [46]. The low nutritional values associated with honey bee and hollyhock pollens indicate low digestibility, possibly explained by high sugar content (glucose and fructose) [45], which is non-nutritive to *H. armigera* larvae. The poor nutritional quality of bee pollen was also demonstrated by Riahi et al. (2016), who reported prolonged developmental time in *Typhlodromus bagdasarjani* Wainstein & Arutunjan, 1967 (Acari: Phytoseiidae) [29]. Larvae fed on a sunflower pollen-based artificial diet exhibited the highest consumption index (CI) and relative consumption rate (RCR), likely due to the high phenolic content, which may reduce the digestibility of the food or its absorption through the inhibition of digestive enzymes. However, increased food consumption did not translate into greater larval or pupal weight, nor did it enhance growth [47]. The heaviest weight gain was observed in larvae fed on a date palm pollen-based diet, whereas a diet without pollen (control) resulted in the lowest weight gain. Truzi et al. (2021) reported that doubling the protein content in a basic artificial diet reduced the larval weight of *Spodoptera frugiperda* (J.E. Smith, 1797) [48], a finding that contradicts the results of the present study. Furthermore, diets containing honey bee, hollyhock, and date palm pollens resulted in significantly higher pre-pupal and pupal weights. Since heavier pupae typically develop into females with greater reproductive potential [49], it is necessary to further evaluate the reproduction of *H. armigera* on these three diets. The increased larval and pupal weights observed in the date palm pollen diet may be attributed to its lower sugar and total phenolic contents compared to other pollen types.

The efficiency of conversion of ingested and digested foods (ECI and ECD, respectively) are key statistical indicators of feeding efficiency and nutritional assimilation among insects and predict weight gain [10]. *Helicoverpa armigera* larvae that were reared on a date palm pollen-based artificial diet exhibited the highest ECI and ECD values. Larvae fed on the date palm pollen diet also displayed the highest relative growth rate (RGR), demonstrating a direct correlation with ECI and ECD values. Conversely, the lowest RGR and corresponding ECI and ECD values were observed among larvae fed on the control diet without pollen. These findings suggest that the date palm pollen-based artificial diet was the most suitable among those tested. Biochemical analysis of the pollen grains suggests that the higher protein content (positive correlation) and lower levels of sugar and total phenolics (negative correlation) in date palm pollen may have contributed to this outcome. Similarly, Goudarzi Mohammadi et al. (2024) reported a negative correlation between phenol content and both ECD and RGR values in *H. armigera* larvae fed on a sunflower seed-based artificial diet [10]. Secondary compounds, such as phenols, act as antifeedants and inhibit normal insect development and growth [50]. Glucose, sucrose, and fructose are the primary sugar types found in pollen grains [29], serving as carbohydrate sources for pollen-feeding insects. However, better nutritional performance of *H. armigera* larvae on pollen grains with lower sugar content suggests that these larvae do not require additional carbohydrates from pollen sugars. Instead, they may obtain necessary carbohydrates from wheat germ or through the breakdown of other macronutrients, such as proteins and lipids. Previous studies have demonstrated that the high nutritional value of almond pollen to *T. bagdasarjani* was due to its high protein and sucrose contents, along with a moderate lipid content [29]. The suitability of pollen as a dietary component for insects depends on several factors, including species-specific nutritional requirements and the biochemical composition of the pollen grains.

The regulation of digestive enzyme activity in insects is a physiological adaptation that allows for dynamic adjustment to changes in food quality and quantity [51]. Our results indicate that a maize pollen-based diet increased α-amylase activity in *H. armigera* larvae. Additionally, the control diet and saffron pollen-based diet enhanced proteolytic activity. Elevated levels of α-amylase and proteases suggest that the primary nutrients in these diets were effectively digested by *H. armigera* larvae. The structure of pollen cell walls plays a crucial role in the digestion of pollen by midgut digestive enzymes [24]. The pollen grains from certain plant species are resistant to enzymatic degradation, leaving their exine largely undigested, whereas others are more susceptible, allowing enzymatic action to create pores in the exine. This facilitates the release of nutrients, particularly amino acids, from the pollen wall into the gut lumen [24,52]. Although cell wall analysis of pollen grains was not conducted in this study, it is likely that the cell walls of maize and saffron pollens were more susceptible to digestive enzymes than those of other pollen types, leading to their increased digestion. Alternatively, it is possible that α-amylases and proteinases are simply the predominant digestive enzymes in the gut of *H. armigera* larvae for carbohydrate and protein metabolism [53].

Typically, higher enzyme activity occurs in response to diets rich in carbohydrates or proteins. However, in this study, maize and saffron pollens contained relatively low levels of both macronutrients. The amounts of macronutrients were also low in certain types of pollen grains, particularly in date palm pollen, while the amylolytic activity of larvae feeding on these diets was high. Similarly, Bidar et al. (2016) reported increased proteolytic activity in *Ephestia kuehniella* Zeller, 1879 when fed a diet with low protein content [54]. This suggests that other carbohydrate and protein sources in the artificial diets, such as wheat germ and cowpea, may have contributed to the enhanced enzyme activity. The results further revealed that proteolytic activity in *H. armigera* was negatively correlated with protein content but positively associated with the total phenolic content of pollen grains. In contrast, amylolytic activity exhibited the opposite trend with total phenolic content. The increased proteolytic activity in larvae fed on the control and saffron pollen-based diets may be a midgut response to the secondary metabolites (such as total phenolics) and/or enzyme inhibitors present in pollen grains or cowpea seeds. Enzyme inhibitors in legume seeds are known to suppress digestive enzyme activity [16]. These inhibitors specifically target proteases by binding to their active sites and forming stoichiometric complexes, ultimately leading to amino acid deficiencies [55]. Consequently, insects may compensate by overproducing digestive enzymes to counteract the inhibitory effects, a mechanism that may have occurred in *H. armigera* larvae [56].

Cluster analysis categorized the pollen-supplemented diets into two distinct groups, designated as Cluster A and Cluster B, based on dietary consumption, nutrient utilization metrics, and digestive enzyme activity in *H. armigera* larvae. Each cluster was further divided into two sub-clusters. Sub-cluster A2 included the most nutritionally favorable diet—the date palm pollen-based formulation—which provided optimal nutritional conditions for *H. armigera* larvae, characterized by a low content of anti-nutritional compounds (e.g., phenolics) and a high concentration of essential nutrients. This was consistent with the high nutritional quality of date palm pollen, noted for its elevated protein content and low phenolic levels, and with superior larval performance indicators, including increased larval weight, weight gain, efficiency of conversion of ingested food (ECI), efficiency of conversion of digested food (ECD), and relative growth rate (RGR). These findings suggest that rearing *H. armigera* on a high-quality nutritional source may lead to improved egg and larval quality, thereby enhancing the effectiveness of parasitoids such as *Trichogramma* and *Bracon* wasps, which rely on these developmental stages for successful parasitism [5]. In contrast, the control diet was placed in sub-cluster B2 and exhibited the lowest nutritional suitability. Diets in sub-clusters A1 and B1 displayed intermediate nutritional and physiological effects, with the honey bee and maize pollen-based diets showing greater similarity to one another compared to the other treatments.

## 5. Conclusions

We evaluated the nutritional efficiency and digestive adaptability of *Helicoverpa armigera* larvae reared on artificial diets supplemented with various pollen types to assess the potential of pollen-enriched diets for mass-rearing purposes. Significant variations in nutritional indices, growth parameters, and digestive enzyme activities were observed among larvae fed the different pollen-containing diets. These physiological changes were closely associated with the biochemical composition of the pollen, particularly the levels of protein and secondary metabolites. Among the tested formulations, the diet supplemented with date palm pollen was identified as the most nutritionally favorable for *H. armigera* larvae. Despite the pollen being incorporated in relatively small quantities, its impact on larval performance was substantial, likely due to its low content of phenolic compounds, which are known to interfere with digestion and nutrient assimilation. These findings suggest that the observed physiological improvements in larvae were primarily influenced by the reduced presence of such secondary metabolites. The date palm pollen-based diet, thus, represents a significant advancement in the formulation of high-quality artificial diets for the efficient mass rearing of *H. armigera*, with promising implications for its use in augmentative biological control programs.

## Figures and Tables

**Table 1:** Compositions of artificial diets containing various pollen grains for rearing *Helicoverpa armigera* larvae.

Ingredient	Unit	Artificial Diet								
		Control	Honey Bee	Rapeseed	Maize	Sunflower	Hollyhock	Glossy-Shower	Saffron	Date Palm
Pollen	g	-	1	1	1	1	1	1	1	1
Cowpea	g	20.5	19.5	19.5	19.5	19.5	19.5	19.5	19.5	19.5
Wheat germ	g	3	3	3	3	3	3	3	3	3
Brewer’s yeast	g	3.5	3.5	3.5	3.5	3.5	3.5	3.5	3.5	3.5
Ascorbic acid	g	0.35	0.35	0.35	0.35	0.35	0.35	0.35	0.35	0.35
Sorbic acid	g	0.11	0.11	0.11	0.11	0.11	0.11	0.11	0.11	0.11
Methyl–p–hydroxy–benzoate	g	0.22	0.22	0.22	0.22	0.22	0.22	0.22	0.22	0.22
Formaldehyde, 37%	mL	0.25	0.25	0.25	0.25	0.25	0.25	0.25	0.25	0.25
Sunflower oil	mL	0.5	0.5	0.5	0.5	0.5	0.5	0.5	0.5	0.5
Agar	g	1.4	1.4	1.4	1.4	1.4	1.4	1.4	1.4	1.4
Distilled water	mL	65	65	65	65	65	65	65	65	65

**Table 2:** Nutritional indices (mean ± SE) of third to fifth instar *Helicoverpa armigera* reared on various pollen-containing artificial diets.

Artificial Diet	CI	AD%	ECI%	ECD%	RCR (mg mg^−1^ day^−1^)	RGR (mg mg^−1^ day^−1^)
Control	4.278 ± 0.074 abc	83.520 ± 0.370 bcd	4.160 ± 0.034 e	4.990 ± 0.039 e	2.172 ± 0.044 e	0.127 ± 0.007 d
Honey bee	4.156 ± 0.075 bc	82.790 ± 0.346 d	6.700 ± 0.221 b	8.100 ± 0.270 b	2.784 ± 0.102 bcd	0.213 ± 0.009 b
Rapeseed	4.060 ± 0.048 bcd	83.050 ± 0.199 cd	5.820 ± 0.126 bcd	7.010 ± 0.158 bcd	2.441 ± 0.066 de	0.150 ± 0.007 cd
Maize	4.113 ± 0.077 bc	83.030 ± 0.355 cd	6.390 ± 0.261 bc	7.700 ± 0.322 bc	2.992 ± 0.118 abc	0.192 ± 0.011 bc
Sunflower	4.605 ± 0.091 a	85.060 ± 0.354 ab	6.030 ± 0.275 bcd	7.100 ± 0.332 bcd	3.454 ± 0.137 a	0.208 ± 0.012 b
Hollyhock	4.303 ± 0.101 ab	81.720 ± 0.406 d	5.390 ± 0.148 cd	6.600 ± 0.186 cd	3.148 ± 0.0156 ab	0.169 ± 0.009 bcd
Glossy shower	4.251 ± 0.057 abc	85.910 ± 0.240 a	5.080 ± 0.087 de	5.910 ± 0.109 d	2.537 ± 0.089 cde	0.139 ± 0.007 d
Saffron	3.895 ± 0.055 cd	84.770 ± 0.426 abc	6.740 ± 0.236 b	7.970 ± 0.297 b	2.397 ± 0.084 de	0.168 ± 0.010 bcd
Date palm	3.702 ± 0.081 d	83.150 ± 0.467 cd	8.430 ± 0.337 a	10.140 ± 0.404 a	2.714 ± 0.043 bcd	0.264 ± 0.012 a

Means followed by different letters in the same column are significantly different (Tukey’s HSD test, *p* < 0.01). CI: consumption index; AD: approximate digestibility; ECI: efficiency of conversion of ingested food; ECD: efficiency of conversion of digested food; RCR: relative consumption rate; RGR: relative growth rate.

**Table 3:** Biochemical characteristics (mean ± SE) of different pollen grains.

Pollen	Protein (%)	Sugar (mg/g)	Lipid (%)	Phenol (µg/g)
Honey bee	16.74 ± 0.01 d	15.830 ± 0.012 b	2.77 ± 0.01 f	60.277 ± 0.015 g
Rapeseed	21.52 ± 0.01 b	8.450 ± 0.012 h	25.80 ± 0.11 a	93.410 ± 0.010 c
Maize	16.66 ± 0.01 e	10.623 ± 0.015 f	12.97 ± 0.01 d	91.083 ± 0.018 d
Sunflower	17.13 ± 0.01 c	13.883 ± 0.018 c	12.70 ± 0.14 d	102.923 ± 0.015 a
Hollyhock	16.68 ± 0.01 de	12.657 ± 0.018 d	17.52 ± 0.01 b	73.147 ± 0.015 f
Glossy shower	15.63 ± 0.01 f	31.353 ± 0.224 a	14.52 ± 0.24 c	99.650 ± 0.012 b
Saffron	9.52 ± 0.01 g	11.233 ± 0.145e	6.71 ± 0.14 e	79.00 ± 0.115 e
Date palm	26.43 ± 0.01 a	9.400 ± 0.012 g	6.38 ± 0.01 e	44.290 ± 0.023 h

Means followed by different letters in the same column are significantly different (Tukey’s HSD test, *p* < 0.01).

**Table 4:** Correlation coefficients (*r*) between the nutritional and physiological characteristics of *Helicoverpa armigera* and the biochemical traits of different pollen grains.

Parameter	Protein	Sugar	Lipid	Total Phenolic
Larval weight	0.339 (0.105)	−0.618 (0.001) *	−0.190 (0.374)	−0.536 (0.007) *
Food consumed	0.246 (0.129)	−0.378 (0.069)	0.143 (0.504)	0.129 (0.548)
Pupal weight	0.363 (0.081)	−0.681 (0.000) *	0.102 (0.392)	−0.535 (0.007) *
ECI	0.524 (0.009) *	−0.588 (0.003) *	−0.361 (0.083)	−0.731 (0.000) *
ECD	0.532 (0.007) *	−0.613 (0.001) *	−0.317 (0.132)	−0.738 (0.000) *
RCR	0.280 (0.184)	−0.116 (0.942)	−0.069 (0.749)	0.040 (0.854)
RGR	0.565 (0.004) *	−0.462 (0.023) *	−0.335 (0.109)	−0.568 (0.004) *
Amylolytic activity	0.219 (0.303)	−0.192 (0.369)	−0.741 (0.000) *	−0.573 (0.003) *
Proteolytic activity	−0.543 (0.006) *	0.213 (0.317)	0.425 (0.038) *	0.494 (0.009) *

The numerals in the parenthesis are *p*-values. Significant correlations are shown with an asterisk. ECI: efficiency of conversion of ingested food; ECD: efficiency of conversion of digested food; RCR: relative consumption rate; RGR: relative growth rate.

## Data Availability

The original contributions presented in this study are included in the article. Further inquiries can be directed to the corresponding author.

## Abbreviations

The following abbreviations are used in this manuscript:

CI	Consumption index
AD	Approximate digestibility
ECI	Efficiency of conversion of ingested food
ECD	Efficiency of conversion of digested food
RCR	Relative consumption rate
RGR	Relative growth rate
